# Editorial of the Special Issue: “Soft and Hard Tissue Regeneration”

**DOI:** 10.3390/biomedicines10020356

**Published:** 2022-02-01

**Authors:** Mike Barbeck, Said Alkildani, Ole Jung

**Affiliations:** 1 Department of Ceramic Materials, Institute for Materials Science and Technologies, Technical University of Berlin, 10587 Berlin, Germany; mike.barbeck@tu-berlin.de; 2 BerlinAnalytix GmbH, 12109 Berlin, Germany; said.alkildani@berlinanalytix.com; 3 Clinic and Policlinic for Dermatology and Venereology, University Medical Center Rostock, 18057 Rostock, Germany

In general, only a total of four tissue classes are distinguished: the covering tissue (epithelial tissue), the connective and supporting tissue (connective tissue, fatty tissue, bone, and cartilage), the muscle tissue, and the nervous tissue. All organs of the body, such as the heart and lungs, as well as functional organ systems such as the skeletal, nervous, and vascular systems, are composed of these four tissue types. Moreover, specialized tissues in the form of organs with dedicated cellular and extracellular organization are known. In total, 12 different organs in the human organism are known. Each organ in the body has a distinctive structure (e.g., heart, lungs, liver, eyes, and stomach) and performs very specific tasks. An organ is composed of different types of tissue and thus also of different types of cells. For example, the heart is made up of muscle tissue that contracts and pumps blood through the veins, fibrous tissue of the heart valves, and special cells that control heart rate and rhythm [1]. In the eye are muscle cells that open and close the pupil, clear cells that form the lens and cornea, cells that produce the fluid contained in the eye, cells that sense light, and nerve cells that conduct impulses to the brain [2]. Even an organ as simple as the gallbladder contains different types of cells, such as those that form a coating of the bladder that protects it from irritation by bile, muscle cells that contract to expel bile, and cells that form the fibrous outer wall that holds the sac together [3].

Various diseases and mainly cancer often threaten the functionality of these various organs, so that in many cases biomaterials or medical devices have to be used in order to be able to restore structure and functionality [4]. In general, materials of both organic and inorganic origin are on the market for a broad variety of applications. For example, these biomaterials include metal alloys, ceramics, polymers, and biocomposites [5,6,7,8]. It quickly becomes apparent how different the basic physicochemical properties of the large heterogeneous group of biomaterials are. Thereby, biomaterials are defined by their function. Since they should replace the function of certain host tissues, they must possess the right respective mechanical, chemical, and biological properties (optimized for their purpose, their specific application, and their respective biological surroundings).

In this context, the term “biocompatibility” is used to describe the appropriate biological requirements of a biomaterial. Its definition describes “biocompatibility” as the ability of a material to perform with an appropriate host response in a specific application [9]. Thus, biocompatibility addresses the identification of a specific host response. Moreover, biocompatibility includes the safety of a biomaterial, which means that a material application does not elicit detrimental local or systemic responses. Therefore, biomaterials have to undergo tissue and animal testing to determine their safety and efficacy prior to human application. For example, the importance of biocompatibility was shown by the consequences of allergic reactions to nickel- and chromium-containing stainless steel implants [10]. In the worst case, the biological rejection of a biomaterial occurs, which can lead to the necessity of its removal (even in combination with biomaterial-induced inflammatory responses). Altogether, a lot of research into new biomaterials is necessary and has to focus on improving biocompatibility of biomaterials.

A variety of biomaterials are currently available on the market for soft and hard tissue regeneration [6,11]. Moreover, a broad spectrum of new materials is currently being developed worldwide. Thus, an enormous number of preclinical in vitro and in vivo studies, and clinical studies as well, are being conducted to clarify the related tissue reactions and their regenerative capacities. The compositions of the biomaterials for soft and hard tissue regeneration vary from natural polymers (such as collagen and decellularized bone matrix) up to synthetic polymers such as polylactid acid (PLA) [12,13]. Furthermore, different new composite materials and metals (e.g., magnesium) are currently being developed and investigated [14]. Moreover, new manufacturing methods such as 3D printing are on the rise and can be of greater interest for future applications [15]. Finally, it is of great interest to conduct further research focusing on the molecular mechanisms of and immunological responses to biomaterials.

In this special issue new insights into the underlying cellular and molecular interactions of biomaterials for hard and soft tissue regeneration are presented ranging from collagen-based matrices for osteoconduction and collagen membranes for guided bone regeneration (GBR) to newly developed methodologies such as electrical stimulation of adipose-derived stem cells, vascular grafts, and bioabsorbable ossification materials for maxillofacial bone surgery [11,16,17,18,19,20,21,22,23]. Altogether, this special issue includes studies describing novel biomaterials and innovative material processing techniques related to the healing processes of soft and hard tissues. 
